# A *Salmonella* Small Non-Coding RNA Facilitates Bacterial Invasion and Intracellular Replication by Modulating the Expression of Virulence Factors

**DOI:** 10.1371/journal.ppat.1002120

**Published:** 2011-09-15

**Authors:** Hao Gong, Gia-Phong Vu, Yong Bai, Elton Chan, Ruobin Wu, Edward Yang, Fenyong Liu, Sangwei Lu

**Affiliations:** 1 Division of Infectious Diseases and Vaccinology, School of Public Health, University of California, Berkeley, California, United States of America; 2 Program in Comparative Biochemistry, University of California, Berkeley, California, United States of America; Stanford University School of Medicine, United States of America

## Abstract

Small non-coding RNAs (sRNAs) that act as regulators of gene expression have been identified in all kingdoms of life, including microRNA (miRNA) and small interfering RNA (siRNA) in eukaryotic cells. Numerous sRNAs identified in *Salmonella* are encoded by genes located at *Salmonella* pathogenicity islands (SPIs) that are commonly found in pathogenic strains. Whether these sRNAs are important for *Salmonella* pathogenesis and virulence in animals has not been reported. In this study, we provide the first direct evidence that a pathogenicity island-encoded sRNA, IsrM, is important for *Salmonella* invasion of epithelial cells, intracellular replication inside macrophages, and virulence and colonization in mice. IsrM RNA is expressed *in vitro* under conditions resembling those during infection in the gastrointestinal tract. Furthermore, IsrM is found to be differentially expressed *in vivo*, with higher expression in the ileum than in the spleen. IsrM targets the mRNAs coding for SopA, a SPI-1 effector, and HilE, a global regulator of the expression of SPI-1 proteins, which are major virulence factors essential for bacterial invasion. Mutations in IsrM result in disregulation of expression of HilE and SopA, as well as other SPI-1 genes whose expression is regulated by HilE. *Salmonella* with deletion of *isrM* is defective in bacteria invasion of epithelial cells and intracellular replication/survival in macrophages. Moreover, *Salmonella* with mutations in *isrM* is attenuated in killing animals and defective in growth in the ileum and spleen in mice. Our study has shown that IsrM sRNA functions as a pathogenicity island-encoded sRNA directly involved in *Salmonella* pathogenesis in animals. Our results also suggest that sRNAs may represent a distinct class of virulence factors that are important for bacterial infection *in vivo*.

## Author Summary

Regulated expression of virulence factors is essential for infection by human pathogens such as *Salmonella*. Small non-coding RNAs (sRNAs) that act as regulators of gene expression have been identified in all kingdoms of life, and many sRNAs in *Salmonella* are encoded by genes located at *Salmonella* pathogenicity islands commonly found in pathogenic strains. In this study, we demonstrated that a pathogenicity island-encoded sRNA directly targets the expression of both a global regulator of virulence genes as well as a specific virulence factor critical for *Salmonella* pathogenesis. The sRNA is important for *Salmonella* invasion of epithelial cells, replication inside macrophages, and virulence/colonization in mice, representing the first example of a pathogenicity island-encoded sRNA that is directly involved in *Salmonella* pathogenesis *in vivo*. Our study suggests that sRNA may function as a distinct class of virulence factors that significantly contribute to bacterial infection *in vivo*. Furthermore, our results raise the possibility of developing new strategies against bacterial infection by preventing the expression of regulatory sRNAs.

## Introduction

*Salmonella* (e.g. *S. enterica* serovars Typhimurium and Enteritidis) is the leading cause of food-borne illnesses in the United States, causing diverse diseases ranging from mild, self-limiting gastroenteritis to life-threatening systemic infection [1]. As a facultative intracellular pathogen, *Salmonella* invades non-phagocytic cells such as intestinal epithelial cells and replicates in phagocytes during systemic infection. Two hallmarks of *Salmonella* pathogenesis, i.e. host invasion and intracellular proliferation, correlate with the genes in *Salmonella* pathogenicity islands (SPIs), which are distinct, relatively large chromosomal regions harboring virulence genes and are commonly found in pathogenic strains [2], [3]. For example, *Salmonella* pathogenicity island 1 (SPI-1) contains invasion genes, while *Salmonella* pathogenicity island 2 (SPI-2) contains genes required for intracellular survival and replication [4], [5], [6]. Both SPI-1 and SPI-2 encode type III secretion systems (T3SS), which are specialized organelles that deliver effector proteins to the cytosol of host cells [3], [7]. The T3SS apparatus is a needle-like structure that spans the inner and outer membranes of the bacterial envelope and penetrates host cell membranes. Through T3SS *Salmonella* secretes translocon proteins that allow the delivery of effector proteins into eukaryotic cells [3], [7], leading to modulation of host cells and immune responses, and promotion of bacterial pathogenesis [4], [5], [6]. Highly regulated expression of the genes in SPIs and those encoding their effector proteins is observed both *in vitro* and *in vivo* and is required for bacterial pathogenesis and infection [6], [8].

The regulation of expression of genes coding for SPI-1 proteins and its effectors is remarkably complex and is yet to be fully characterized [8]. For example, the expression of some genes coding for SPI-1 proteins, including SopA, has been shown to be induced upon invasion of both macrophages and epithelial cells and at the late stage of *Salmonella* infection in animals [9], [10], [11], [12], [13]. These results suggest that in addition to their generally recognized roles in invasion, the SPI-1 factors may play an important role post-invasion, possibly in intracellular replication/survival.

Small non-coding RNAs (sRNAs) that act as regulators of gene expression have been identified in all kingdoms of life, including microRNA (miRNA) and small interfering RNA (siRNA) in eukaryotic cells [14], [15], [16], [17]. The bacterial sRNAs are generally untranslated, and range from 50–300 nucleotides in length. They play diverse physiological roles such as those in the regulation of stress responses and metabolism, and control of bacterial envelope composition [16], [18], [19]. The mechanisms by which bacterial sRNAs modulate gene expression are diverse, and two general modes of action have been established, dividing regulatory sRNAs into two classes [16], [18], [19], [20]. One class of sRNAs acts by interacting with a protein target to modify its activity [21], [22]. The other class of sRNAs acts by base-pairing with one or more target RNAs. Most of these antisense RNAs base-pair with target mRNAs in trans with partial complementarity to modulate their translation and/or stability [16], [17], [20].

While many protein factors (e.g. SPI-1 and SPI-2 factors) are shown to be important for bacterial pathogenesis, little is known about the functions of sRNAs in *Salmonella* virulence. Recently novel sRNA genes have been identified within the SPIs [23], [24], [25]. The expression of many of these sRNA genes is associated with stress conditions and the stationary phase [25]. In addition, several SPI-encoded sRNAs were found to be expressed in *Salmonell*a that resides within macrophages [23]. The location of these sRNA genes in the SPIs suggests that these sRNAs may play important roles in bacterial pathogenesis and virulence.

Here we demonstrate that a SPI-encoded sRNA, IsrM sRNA, modulates the differential expression of SPI-1 genes by independently targeting the mRNAs of both the global SPI-1 regulator HilE and specific SPI-1 effector SopA. *Salmonella* with mutations in *isrM* is defective in bacterial invasion and intracellular replication *in vitro*, and in virulence and colonization in infected mice. To our knowledge, IsrM is the first pathogenicity island-encoded RNA known to be important in *Salmonella* pathogenesis *in vivo*. Our study suggests that sRNAs may represent a distinct class of virulence factors that significantly contribute to bacterial pathogenesis *in vivo*.

## Results

### Expression of IsrM RNA *in vitro* under conditions resembling those of the gastrointestinal tract

**Figure 1:**
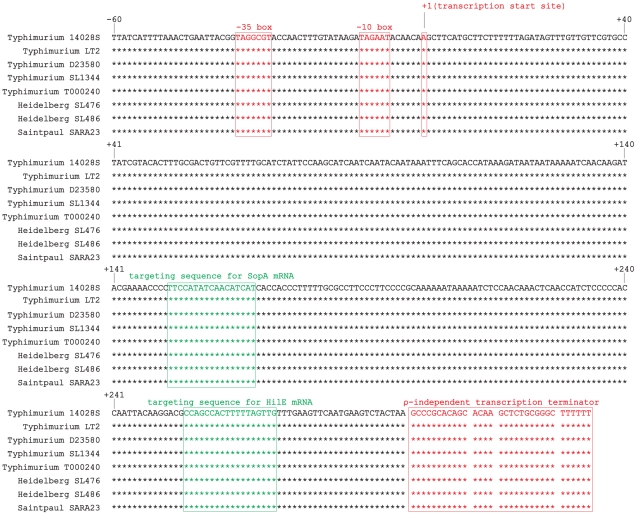
Alignment of *isrM* sequences of different *Salmonella* strains including the upstream promoter region, based on BLAST searches of the IsrM sequences in GenBank. The transcription start sites, −10 boxes, −35 boxes, and transcription terminators of isrM genes are boxed and in red [23]. The targeting sequences for SopA and HilE mRNAs that were identified in this study are boxed and in green.

Because IsrM is a pathogenicity island-specific sRNA of *Salmonella* and is not found in *E.coli* [23], it is conceivable that IsrM plays a role in pathogenesis of *Salmonella*. The gene encoding IsrM can be found in several serovars of *Salmonella* such as *Salmonella typhimurium* but not other serovars such as *Salmonella typhi or Samonella bongori* (Figure 1). It is currently unclear why IsrM is found in some serovars but not other serovars of *Salmonella*, although it is possible that the presence of IsrM in a specific serovar may be related to the unique pathogenesis of the serovar in a particular host. IsrM is expressed as a 329-nucleotide RNA at both the logarithm **(**log) and stationary growth phases, and is expressed at a moderate level in *Salmonella* isolated from J774 macrophages [23]. However, little is currently known about the expression of IsrM *in vitro* under conditions resembling those at early stages of infection, and its expression during *Salmonella* infection *in vivo* has not been reported.

**Figure 2:**
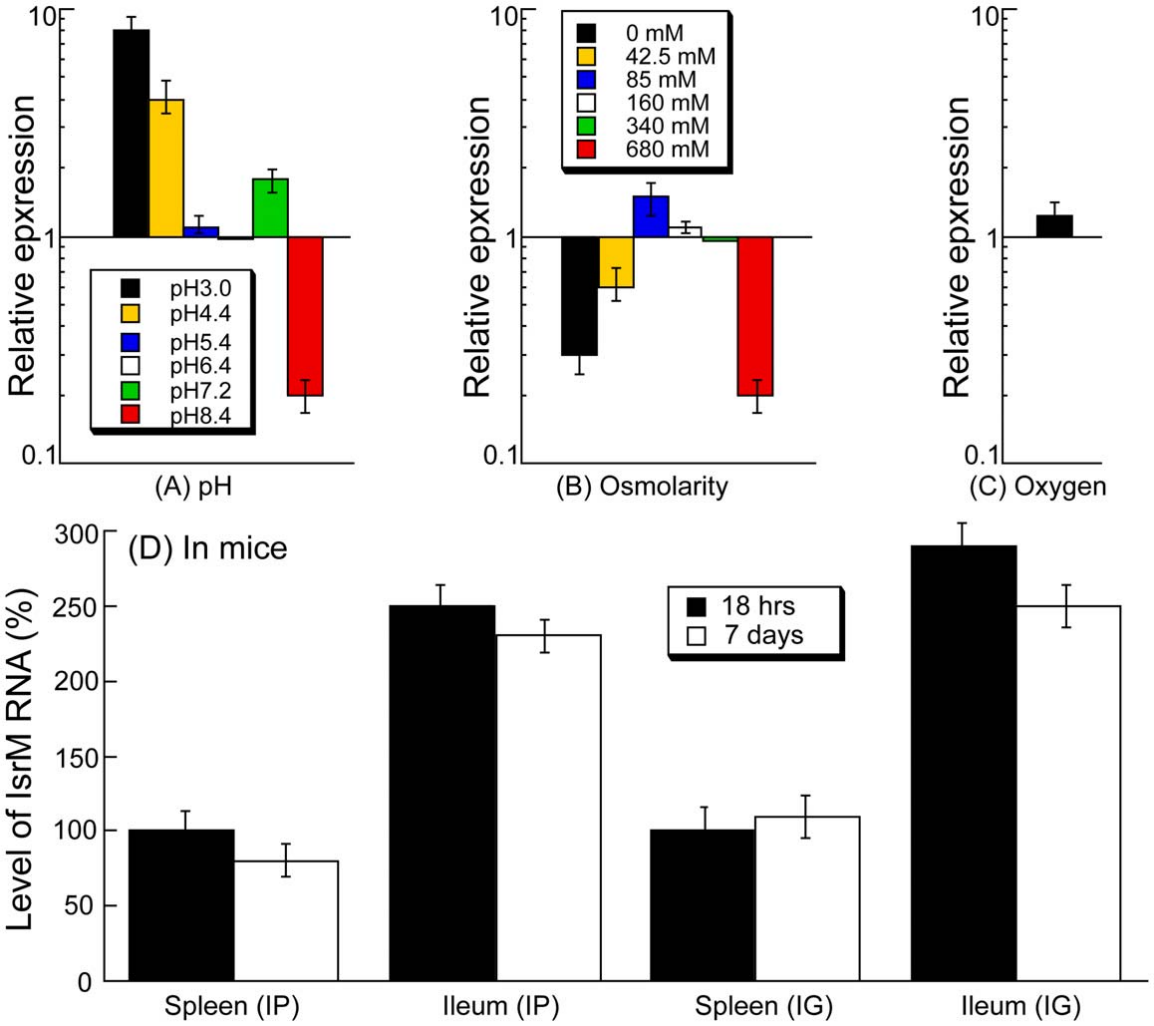
Expression of IsrM RNA in *Salmonella*. *Salmonella* was grown either *in vitro* (A–C) or in mice (D). Effect of pH values (A), osmolarity (B), and limitation of oxygen (C). Cultures of ST14028s were grown in culture media at different pH, at various concentrations of NaCl, or in the presence and limitation of oxygen. The values of the relative expression, which are the means from triplicate experiments, represent the ratios of the levels of IsrM under each pH condition to that under the control pH7.0 condition (A), the ratios of the levels of IsrM under different osmolarity conditions to that under the control condition of 170 mM NaCl as in regular LB broth (B), or the ratios of the level of IsrM under oxygen limitation to that under the control condition (i.e. in the presence of oxygen) (C). The analyses were repeated three times. The standard deviation is indicated by the error bars. (D) Level of IsrM from *Salmonella* recovered from the spleen and ileum of infected mice. SCID mice were intraperitoneally (IP) or intragastrically (IG) infected with ST14028s, and bacteria were recovered from the organs at 18 hours or 7 days post inoculation, respectively. The values in the IP and IG experiments, which are the means from triplicate experiments, represent the relative level of IsrM in ST14028s recovered from the spleen and ileum, as compared to the level of IsrM in *Salmonella* recovered from the spleen of the IP- and IG-infected mice at 18 hours post inoculation, respectively.

During natural infection via the oral route, *Salmonella* encounters a series of environmental changes in the gastrointestinal track that prime *Salmonella* for invasion of the intestinal epithelium [26], [27], [28], [29], [30], [31], [32]. To study the role of IsrM in *Salmonella* invasion, we exposed *Salmonella* to three different stimuli that resembled those *Salmonella* likely encounters during the early stages of its oral infection: pH, osmolarity, and oxygen limitation. The expression of IsrM was examined using quantitative RT-PCR (qRT-PCR) assays with the level of 16S rRNA as the internal control (Figure 2A–C).

During enteric infection, *Salmonella* needs to survive in the stomach, which is acidic, before establishing colonization in the intestine, which is relatively basic. Quantitative RT-PCR assays showed that IsrM had higher expression at pH3.0, pH4.4, and pH7.2, with the highest expression at pH3.0, compared to that at pH7.0 (Figure 2A). These results were further confirmed using Northern blot analyses. For example, using the level of 5S RNA as the internal control, Northern analyses showed that the level of IsrM was higher at pH4.4 than at pH7.0 (Figure S1)(Supporting Information). These observations suggest that this sRNA may be expressed as early as in the stomach during *Salmonella* infection *in vivo*. High osmolarity is an environmental stress that bacteria encounters in the intestine, and is believed to promote *Salmonella* adhesion and invasion of intestinal epithelial cells, contributing to its virulence [28]. Increasing osmolarity up to 85 mM NaCl favored the expression of IsrM (Figure 2B). Oxygen limitation, an important characteristic of the environment in the intestine, can induce *Salmonella* invasiveness of intestinal mucosa while aerobic conditions render *Salmonella* less invasive [29], [30], [31], [32]. In our experiments, IsrM expression was moderately induced by oxygen limitation (Figure 2C). Thus, IsrM RNA was apparently expressed under conditions that resembled the early stages of its oral infection in the gastrointestinal tract.

### Expression of IsrM during *Salmonella* infection *in vivo*

Both immunocompetent BALB/c mice and immunodeficient SCID mice were infected intraperitoneally to study IsrM expression during systemic bacterial infection. Immunodeficient animals, such as the CB17 SCID mice that lack functional T and B lymphocytes, are extremely susceptible to *Salmonella* infection [12], [13]. Analysis of bacterial growth in these mice can be used for comparing the virulence and studying the pathogenesis of different bacterial strains and mutants [12], [13]. At different time points postinfection, the spleen and ileum were harvested. Quantitative RT-PCR was carried out to determine the expression of IsrM RNA in *Salmonella* isolated from the tissues, using the expression of bacterial 16S rRNA as the internal control (Figure 2D). Normalization of samples was also carried out by using total RNAs extracted from the same colony forming unit (CFU) (e.g. 5×10^4^ CFU) of bacteria. Similar amounts of 16S rRNA were detected from same CFU *Salmonella* regardless of infection route (intraperitoneally or intragastrically) or time point postinfection (12–24 hours or 5–7 days) (data not shown), suggesting that the level of 16S rRNA was not significantly different in bacteria from the spleen and ileum. *Salmonella* presumably trafficked to the ileum tissue via the lymphatic system in intraperitoneally-infected mice, while in intragastrically-infected mice, the bacteria could directly invade and infect the ileum tissue.

*Salmonella* isolated from both the spleen and ileum of SCID mice at 18 hours postinfection were found to express IsrM, with its expression in the ileum approximately 2.5-fold higher than that in the spleen (Figure 2D). To rule out the residual expression of IsrM from *in vitro* bacterial growth, mice were intraperitoneally infected with bacteria for a longer period and tissues were harvested at 7 days postinfection. We also found that IsrM was expressed in both organs, with its expression in the ileum approximately 2.5-fold higher than that in the spleen. Similar results were also observed in bacteria isolated from the organs of the intraperitoneally-infected BALB/c mice (data not shown).

SCID mice were also infected intragastrically with bacteria in order to study whether IsrM is expressed during *Salmonella* infection acquired by the oral route. Spleens and ileums were collected and the bacteria were recovered at 18 hrs and 7 days postinfection. IsrM was detected in *Salmonella* isolated from both the spleen and ileum, with its level in the ileum approximately 2.5-fold higher than that in the spleen (Figure 2D). Similar results were also observed in *Salmonella* isolated from the organs of infected BALB/c mice. Taken together, these results suggest that IsrM is expressed during the early and late stages of *Salmonella* infection, and that it is differentially expressed, with higher expression in the ileum than in the spleen.

### IsrM is dispensable for *Salmonella* growth *in vitro* but important for the expression of *Salmonella* SPI-1 proteins

To investigate the role of IsrM in *Salmonella* growth, a mutant, ΔIsrM, was derived from the wild type *S. typhimurium* 14028s strain by deleting the IsrM sequence. Mutant ΔIsrM grew as well as ST14028s in LB broth (Figure S2A)(Supporting Information), indicating that IsrM is dispensable for bacterial growth *in vitro*.

The unique presence of IsrM in a SPI region and its induced expression in the ileum suggests that IsrM may play a role in the pathogenesis of *Salmonella* in the intestine. Since SPI-1 proteins are major virulence determinants essential for *Salmonella* invasion [3], [7], we reasoned that IsrM may function to regulate the expression of SPI-1 factors, facilitating *Salmonella* infection and pathogenesis. To investigate this possibility, we examined the effect of a deletion of the IsrM sequence on the expression of SPI-1 factors. Bacterial strains T-invJ, T-sopE2, T-sptP, T-spaO, T-sipD, T-sipC, T-sipB, T-sipA, T-sopB, and T-sopA were previously derived from the wild type *Salmonella* strain ST14028s by inserting the FLAG epitope tag sequence into SPI-1 ORFs *invJ, sopE2*, *sptP*, *spaO*, *sipD*, *sipC*, *sipB*, *sipA*, *sopB*, and *sopA*, respectively [12], [13]. Tagging of the SPI-1 ORFs in these mutants did not impair the invasiveness, growth, or the virulence of the bacteria, and the tagged strains can be used as model strains to study the infection of *Salmonella in vitro* and *in vivo*, including the expression of SPI-1 proteins and effectors [12], [13]. We constructed mutants ΔM-T-invJ, ΔM-T-sopE2, ΔM-T-sptP, ΔM-T-spaO, ΔM-T-sipD, ΔM-T-sipC, ΔM-T-sipB, ΔM-T-sipA, ΔM-T-sopB, and ΔM-T-sopA from T-invJ, T-sopE2, T-sptP, T-spaO, T-sipD, T-sipC, T-sipB, T-sipA, T-sopB, and T-sopA, respectively, by deleting the IsrM sequence in each SPI-1 tagged strain (Table 1). These newly constructed *isrM*-deletion mutant strains grew as well as the parental strains and the wild type ST14028s in LB broth (Figure S2B)(Supporting Information), consistent with our results (Figure S2A)(Supporting Information) that IsrM is not essential for bacterial growth *in vitro*.

**Table 1:** Bacterial strains used in this study.

Bacterial strains	Description	Reference/source
ST14028S	Wild type and parental strain	[50]
ΔIsrM-P	ST14028S *isrM*::kan, polar strain	This study
ΔIsrM	ST14028S, *isrM* deleted, without kanR	This study
T-invJ	ST14028S *invJ*::1xFLAG	[13]
ΔM-T-invJ	ST14028S *invJ*::1xFLAG isrM::kanR	This study
T-sipA	ST14028S *sipA*::1xFLAG	[12]
ΔM-T-sipA	ST14028S *sipA*::1xFLAG isrM::kanR	This study
T-sipB	ST14028S *sipB*::1xFLAG	[12]
ΔM-T-sipB	ST14028S *sipB*::1xFLAG isrM::kanR	This study
T-sipC	ST14028S *sipC*::1xFLAG	[13]
ΔM-T-sipC	ST14028S *sipC*::1xFLAG isrM::kanR	This study
T-sipD	ST14028S *sipD*::1xFLAG	[13]
ΔM-T-sipD	ST14028S *sipD*::1xFLAG isrM::kanR	This study
T-sopA	ST14028S *sopA*::1xFLAG	[13]
ΔM-T-sopA	ST14028S *sopA*::1xFLAG isrM::kanR	This study
T-sopB	ST14028S *sopB*::1xFLAG	[13]
ΔM-T-sopB	ST14028S *sopB*::1xFLAG isrM::kanR	This study
T-sopE2	ST14028S *sopE2*::1xFLAG	[12]
ΔM-T-sopE2	ST14028S *sopE2*::1xFLAG isrM::kanR	This study
T-spaO	ST14028S *spaO*::1xFLAG	[12]
ΔM-T-spaO	ST14028S *spaO*::1xFLAG isrM::kanR	This study
T-sptP	ST14028S *sptP*::1xFLAG	[12]
ΔM-T-sptP	ST14028S *sptP*::1xFLAG isrM::kanR	This study
T-hilE	ST14028S *hilE*::1xFLAG	This study
ΔM-T-hilE	ST14028S *hilE*::1xFLAG isrM::kanR	This study
*E coli* Top10	F- *mcrA* Δ(*mrr-hsdRMS*-*mcrBC*) ϕ′*80lacZ*Δ*M15* Δ*lacX74 recA1 araD139* Δ(*ara-leu*)7697 *galU galK rpsL* (*StrR*) *endA1 nupG*	Invitrogen
*E coli* Top 10 F′	F′{*lacIq Tn10 (TetR)} mcrA Δ(mrr-hsdRMS-mcrBC) Φ80lacZΔM15 ΔlacX74 recA1 araD139 Δ(ara-leu)7697 galU galK rpsL endA1 nup*G	Invitrogen

**Figure 3:**
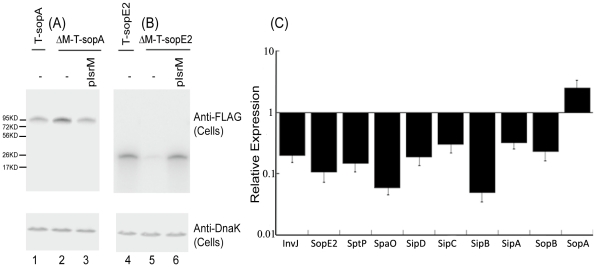
Expression of the tagged proteins in *Salmonella*. (A–B) Western blot analyses of the expression of the tagged proteins from bacterial strains T-sopA and ΔM-T-sopA (A), and T-sopE2 and ΔM-T-sopE2 (B). The expression of bacterial DnaK was used as the internal control. Protein samples were separated in SDS-polyacrylamide gels and reacted with antibodies against the FLAG sequence and DnaK. Each lane was loaded with lysate prepared from 5×10^7^ CFU bacteria. (C) The effect of the deletion of the *isrM* sequence on the expression of SPI-1 proteins. The values of the relative expression, which are the means of triplicate experiments, represent the ratios of the levels of the tagged proteins in ΔM-T-invJ, ΔM-T-sopE2, ΔM-T-sptP, ΔM-T-spaO, ΔM-T-sipD, ΔM-T-sipC, ΔM-T-sipB, ΔM-T-sipA, ΔM-T-sopB, and ΔM-T-sopA to those in T-invJ, T-sopE2, T-sptP, T-spaO, T-sipD, T-sipC, T-sipB, T-sipA, T-sopB, and T-sopA, respectively.

We performed Western analyses to determine the expression of the tagged SPI-1 proteins with an anti-FLAG antibody, using the expression of bacterial DnaK protein as the internal control (Figure 3A). Normalization of samples was also carried out by loading total protein extracted from the same CFU (e.g. 5×10^7^ CFU) of bacteria in each lane. The levels of the tagged InvJ, SpoE2, SptP, SpaO, SipD, SipC, SipB, SipA, and SopB proteins in the IsrM deletion strains were found to be lower than those in the parental strains (Figure 3). In contrast, the protein level of SopA in the IsrM-deletion strain (i.e. ΔM-T-sopA) was found to be approximately 2.5-fold higher than that in the parental T-sopA strain (Figure 3A, compare lanes 1 and 2). Transformation of plasmid pIsrM, which contained the sequence of IsrM under its native promoter and transcriptional terminator sequence, in the deletion mutants restored the levels of the tagged proteins to the wildtype levels (Figure 3, lanes 3 and 6). Using 16S rRNA as the internal control, qRT-PCR assays showed that the levels of IsrM were similar in the parental strain T-sopA and the deletion mutant ΔM-T-SpoA that contained the complementation construct pIsrM (data not shown). Taken together, these results suggest that deletion of IsrM may lead to a global disregulation of expression of the SPI-1 proteins.

### Targeting the 5′ untranslated region (UTR) of the HilE mRNA by IsrM

**Figure 4:**
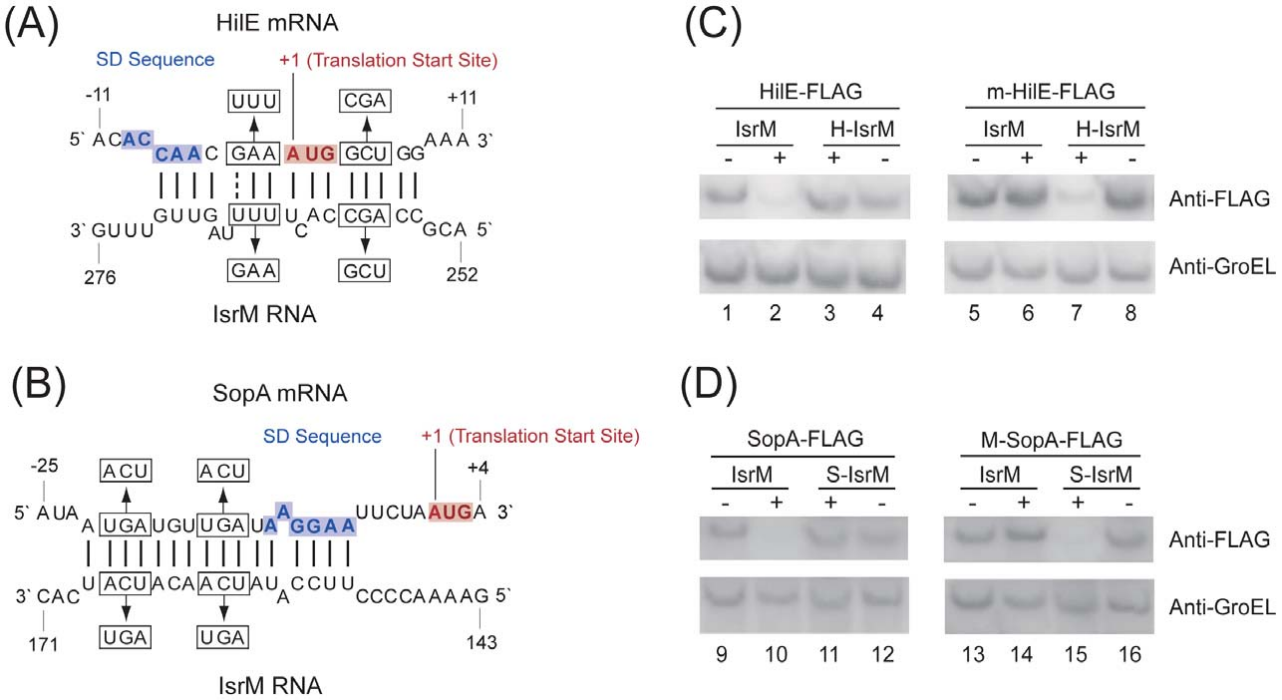
Post-transcriptional targeting of *hilE* and *sopA* by IsrM. (A–B) Schematic representation of the proposed interactions of IsrM sRNA to *hilE* (A) and *sopA* mRNAs (B), and of compensatory base-pair changes. Numbers indicate relative position to the translational start site of *hilE* and *sopA* or position downstream of the transcriptional start site of *isrM*. Arrows denote nucleotide substitutions (in box) introduced to IsrM, and *hilE* and *sopA* mRNAs. The SD and AUG sequences are highlighted in blue and red, respectively. (C–D) Western blot analysis of *E. coli* carrying pLaco-IsrM, pLaco-H-IsrM and pLaco-S-IsrM, in combination with either wild type or mutant target plasmids, as indicated. The expression of the tagged HilE (C) and SopA (D) proteins was determined using that of GroEL as the internal control.

Bacterial sRNAs generally function to modulate gene expression by hybridizing to the target mRNAs [18], [19], [20]. No targets for IsrM have been reported. Using the *TargetRNA* and *RNAhybrid* algorithms, we searched the regions of *Salmonella* mRNAs for potential RNA duplex formation with IsrM [33], [34]. This analysis predicted stable pairing of two distinct and non-overlapping regions of IsrM with the entire Shine-Dalgarno (SD) sequence of *hilE* and *sopA* mRNAs, respectively (Figure 4A–B), which may cause translational repression [18], [19], [20].

HilE is a negative global regulator of the expression of many SPI-1 genes by sequestering HilD, a major SPI-1 transcriptional activator [8], [35]. To determine if IsrM regulates *hilE*, we constructed a tagged *hilE* strain T-hilE by inserting the FLAG epitope tag sequence into *hilE* of ST14028s and furthermore, a corresponding *isrM* deletion mutant of T-hilE, ΔM-T-hilE (Table 1). Both T-hilE and ΔM-T-hilE grew as well as ST14028s in LB broth, and tagging of *hilE* did not impair the invasiveness, growth, or the virulence of the bacteria in mice (data not shown).

**Figure 5:**
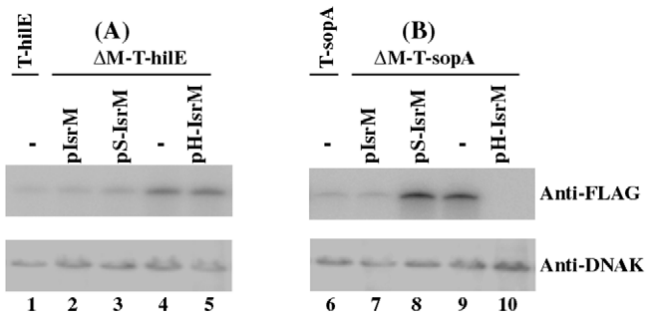
Western blot analysis of the expression of the tagged proteins from *Salmonella*. The tagged proteins were expressed from strains T-hilE and ΔM-T-hilE (A), and T-sopA and ΔM-T-sopA (B). The expression of bacterial DnaK was used as the internal control. Protein samples were separated in SDS-polyacrylamide gels and reacted with antibodies against the FLAG sequence and DnaK. Each lane was loaded with lysate prepared from 5×10^7^ CFU bacteria.

Using the expression level of DnaK as the internal control, Western blot analyses with an anti-FLAG antibody indicated that deletion of IsrM increased the level of HilE (Figure 5, compare lane 1 and 4). Transformation of plasmid pIsrM carrying the IsrM sequence with the native promoter and transcriptional terminator reduced the HilE expression to the wildtype level in ΔM-T-hilE (Figure 5, lane 2), suggesting that IsrM specifically modulates the protein level of HilE.

To investigate the potential interactions between IsrM and *hilE* mRNA, we used a two plasmid system [36] that involved co-expression of the sRNA and a translational target gene with the FLAG fusion at its carboxyl terminus. By transforming both plasmids in *E.coli*, we tested whether the sRNA expression directly affects the protein level of the FLAG-tagged target gene. The 5′ UTR region and the entire coding sequence of *hilE* were cloned into a *FLAG* fusion vector. Transcription of the HilE-FLAG fusion was driven by the constitutive PLtetO-1 promoter to specifically assay post-transcriptional regulation. The IsrM sequence was cloned under the control of the IPTG-inducible PLaco promoter, yielding construct pLaco-IsrM. *E.coli* transformed with both the *hilE*::*flag* fusion and pLaco-IsrM plasmid was grown and split into two cultures, only one of which was treated with IPTG. IsrM-dependent regulation was determined by Western blot analysis of the HilE-FLAG expression (Figure 4C). Our results showed that the expression of IsrM significantly reduced HilE-FLAG expression (Figure 4C, lanes 1 and 2).

To confirm the predicted base-pairing interactions between *hilE* mRNA and IsrM, point mutations were introduced to *isrM* (U_264_U_265_U_266_ ->A_264_A_265_G_266_) and *hilE* (G_-3_A_-2_A_-1_ ->U_-3_U_-2_U_-1_)to generate mutant H-IsrM and m-HilE (Figure 4A). The expression of HilE-FLAG was not repressed by mutant H-IsrM (Figure 4C, lanes 3 and 4), while IsrM did not significantly affect the expression of mutant m-HilE-FLAG (lanes 5 and 6). Repression of HilE expression by IsrM was restored using m-HilE-FLAG and H-IsrM, which contained the compensatory mutations (lanes 7 and 8). To determine the degree of IsrM over-expression from the plasmids used and to correlate the level of IsrM expression with the levels of HilE protein, qRT-PCR assays were performed to determine the level of IsrM. Table S1 (Supporting Information) shows the relative levels of IsrM and the respective levels of HilE protein in the experiments. These results suggest that no significant difference in the expression levels of IsrM in the presence of IPTG was found in these experiments, and that base-pairing interactions between the specific sequences of IsrM (U_264_U_265_U_266_) and HilE mRNA (G_-3_A_-2_A_-1_) were required for IsrM-mediated downregulation of HilE protein expression.

To further validate the interactions between IsrM and the *hilE* mRNA, an additional pair of compensatory mutations was introduced to *isrM* (A_257_G_258_C_259_ ->U_257_C_258_G_259_) and *hilE* (G_4_C_5_U_6_ ->C_4_G_5_A_6_) to generate mutants H2-IsrM and m2-HilE, respectively (Figure 4A). Repression of the m2-HilE-FLAG protein was restored only by using H2-IsrM, which contained compensatory mutations (data not shown). These results suggest that IsrM inhibits HilE expression by binding to its mRNA around the SD sequence.

### Targeting the 5′ UTR of the SopA mRNA by IsrM

Similar experiments were performed to validate the potential interactions between IsrM and the *sopA* mRNA. Since the transcription initiation site of the *sopA* mRNA has not definitively been determined, the sequence between the position 61 nt upstream the translation initiation codon and the position coding for the 258^th^ amino acid residue of SopA, which covered the sequence predicted to interact with IsrM, was cloned into the PLtetO-1-*FLAG* fusion vector. *E.coli* transformed with both the *sopA*::*flag* fusion and pLaco-IsrM plasmid was grown and split into two cultures, only one of which was treated with IPTG. Western blot analysis indicated that the expression of IsrM significantly reduced SopA-FLAG expression (Figure 4D; lanes 9 and 10). To confirm the predicted potential base-pairing interactions between IsrM and the *sop*A mRNA, point mutations were introduced to *isrM* (U_165_C_166_A_167_ ->A_165_G_166_U_167_) and *sopA* (U_-21_G_-20_A_-19_ ->A_-21_C_-20_U_-19_) to generate mutant S-IsrM and M-SopA (Figure 4B). The expression of SopA-FLAG was not repressed by S-IsrM (Figure 4D, lanes 11 and 12), while IsrM did not significantly affect the expression of M-SopA-FLAG (lanes 13 and 14). Repression of M-SopA-FLAG expression was restored by using S-IsrM, which contained the compensatory mutations (lanes 15 and 16). To determine the degree of IsrM over-expression from the plasmids used and to correlate the level of IsrM expression with the levels of SopA protein, qRT-PCR assays were performed to determine the level of IsrM. Table S2 (Supporting Information) shows the relative levels of IsrM and the respective levels of SopA protein in the experiments. These results suggest that no significant difference in the expression levels of IsrM in the presence of IPTG was found in these experiments, and that base-pairing interactions between the specific sequences of IsrM (U_165_C_166_A_167_) and SopA mRNA (U_-21_G_-20_A_-19_) were required for IsrM-mediated downregulation of SopA protein expression.

To further validate the interactions between IsrM and *sopA* mRNA, an additional pair of compensatory mutations was introduced to *isrM* (U_159_C_160_A_161_ ->A_159_G_160_U_161_) and *sopA* (U_-15_G_-14_A_-13_ ->A_-15_C_-14_U_-13_) to generate mutants S2-IsrM and M2-sopA, respectively (Figure 4B). Repression of the M2-SopA-FLAG protein was restored only by using S2-IsrM, which contained compensatory mutations (data not shown). Taken together, these results suggest that IsrM inhibits SopA expression by binding to its mRNA around the SD sequence.

### Independent targeting of *hilE* and *sopA* mRNAs by IsrM in *Salmonella*

Since HilE globally regulates the expression of most of SPI-1 proteins including SopA [8], targeting of either *hilE* or *sopA* mRNAs by IsrM is expected to modulate the SopA protein expression. Consistent with this notion, the expression of SopA in the *isrM* deletion mutant ΔM-T-SopA was suppressed to the wildtype level only by transformation of plasmid pIsrM (Figure 5, lane 7), which contained the wild type full length IsrM sequence, but not by pS-IsrM, which contained mutated IsrM sequence at the SopA binding site with nucleotide substitutions (U_165_C_166_A_167_ ->A_165_G_166_U_167_) (Figure 5, lane 8). The over-expression of SopA in ΔM-T-SopA was also suppressed by transformation of pH-IsrM (Figure 5, lane 10), which contained mutations in IsrM sequence that disrupted the binding of IsrM to *hilE* mRNA (U_264_U_265_U_266_ ->A_264_A_265_G_266_). Interestingly, the SopA level was lower in the pH-IsrM transformed ΔM-T-SopA than the pIsrM transformed mutant (compare lane 10 to lane 7 of Figure 5). One possible explanation for the observation is that pIsrM suppresses HilE while pH-IsrM does not due to mutations in the *hilE* mRNA binding site, and therefore the HilE level in the pH-IsrM transformed mutant is likely higher than in the pIsrM transformed mutant (compared lane 5 and 2 of Figure 5). Since HilE negatively regulates SopA and other SPI-1 factors, the higher HilE level in the pH-IsrM transformed mutant may lead to further suppression of SopA as compared to the pIsrM transformed mutant. The expression of tagged HilE protein could also be reduced to the wildtype level in the *isrM* deletion mutant ΔM-T-hilE by construct pIsrM or pS-IsrM with mutations in the binding site to *sopA* mRNA, but not with construct pH-IsrM that contained mutations in the binding site to *hilE* mRNA (Figure 5A). Quantitative RT-PCR assays showed no significant differences in the expression levels of the wild type and mutant IsrM between *Salmonella* carrying complementation constructs pIsrM, pH-IsrM, or pS-IsrM (data not shown). These observations suggest that IsrM targets HilE and SopA independently by using different regions to bind to these mRNAs separately.

### Role of IsrM in *Salmonella* invasion

**Figure 6:**
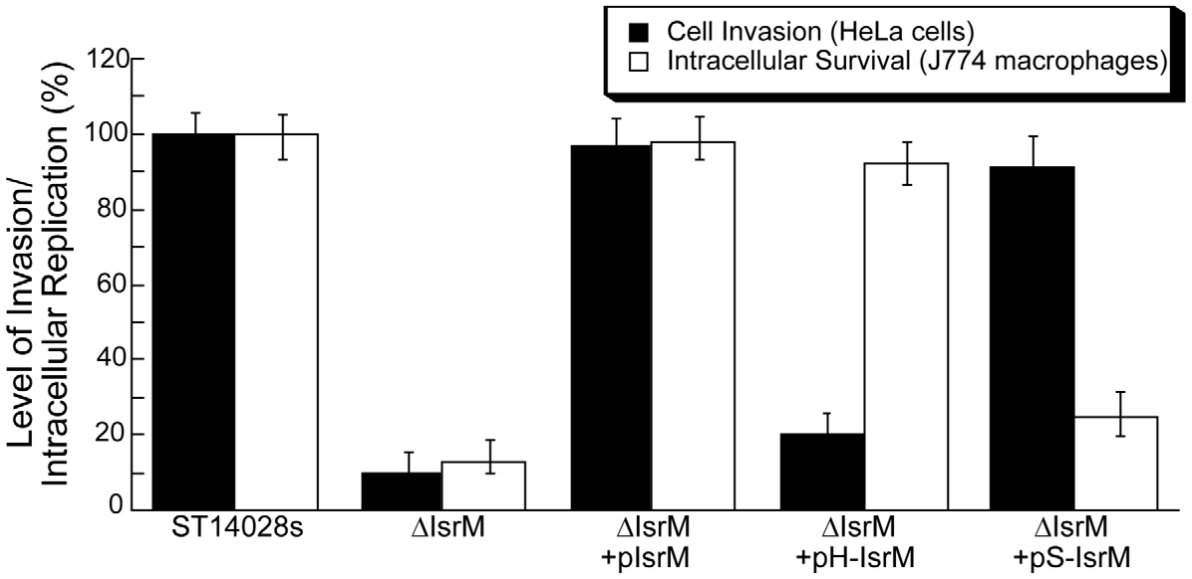
Epithelial cell invasion and intracellular replication in macrophages. Isogenic *Salmonella* strains carrying different constructs were added to HeLa cells at a MOI of 10, and cell invasion was assayed by determining the ratio of the number of intracellular bacteria at 1 hour postinfection to the number of input bacteria. The ratio for wild-type strain ST14028s at 1 h postinfection was arbitrarily defined as 100%, and the ratios for other samples were expressed as relative values. For the intracellular replication assay, *Salmonella* was added to J774 macrophages at a MOI of 10. Intracellular growth was assayed by determining the ratio of the number of intracellular bacteria at 8 hour postinfection to the initial number of bacteria at time zero. The ratio for wild-type strain ST14028s at 8 h postinfection was arbitrarily defined as 100%, and the ratios for other samples were expressed as relative values. The data are the averages of three experiments performed in triplicate. The error bars indicate standard deviations.

It is expected that mutations at IsrM, which result in altered expression levels of HilE, SopA, and other SPI-1 proteins, affect *Salmonella* invasion since proper expression of SPI-1 factors is required for *Salmonella* entry to nonphagocytosed cells. To determine whether this is the case, we tested the ability of various mutant *Salmonella* strains to invade cultured epithelial cells. A reduction of about 90% in invasion of HeLa cells was observed in ΔIsrM, compared to the wild type ST14028s (Figure 6). Transformation with the wild type complementation construct pIsrM restored the ability of ΔIsrM to invade (Figure 6). Furthermore, the level of invasion of ΔIsrM could be restored by more than 90% with transformation of construct pS-IsrM, which expressed a mutated IsrM (S-IsrM) with mutations in the binding site to the *sopA* mRNA, but not with construct pH-IsrM, which expressed a mutated IsrM (H-IsrM) with mutations in the binding site to the *hilE* mRNA (Figure 6). These results suggest that IsrM is important for bacterial invasion by modulating the expression of HilE protein.

### Role of IsrM in intracellular survival in macrophages

Intracellular replication in macrophages represents a major aspect of *Salmonella* pathogenesis [4], [5], [6]. The ability of various strains to proliferate intracellularly was assayed in mouse J774 macrophages [37]. A reduction of about 85% in intracellular replication was observed in ΔIsrM, compared to the wild type ST14028s (Figure 6). Transformation with the wild type complementation construct pIsrM fully restored the ability of ΔIsrM to replicate in macrophages, while the ability of ΔIsrM to replicate in J774 cells was also restored by about 80% by transformation with construct pH-IsrM that expressed the *hilE* mRNA binding-defective IsrM mutant (H-IsrM), but not with construct pS-IsrM that expressed the *sopA* mRNA binding-defective IsrM mutant (S-IsrM) (Figure 6). Meanwhile, these strains, which carried different complementation constructs, appeared to have no growth defect *in vitro* as they replicated as well as the wild type ST14028s strain in LB (Figure S2)(Supporting Information). These results suggest that IsrM is important for intracellular proliferation of *Salmonella* in macrophages by modulating the expression of SopA protein.

### Role of IsrM in bacterial pathogenesis and virulence in mice

**Figure 7:**
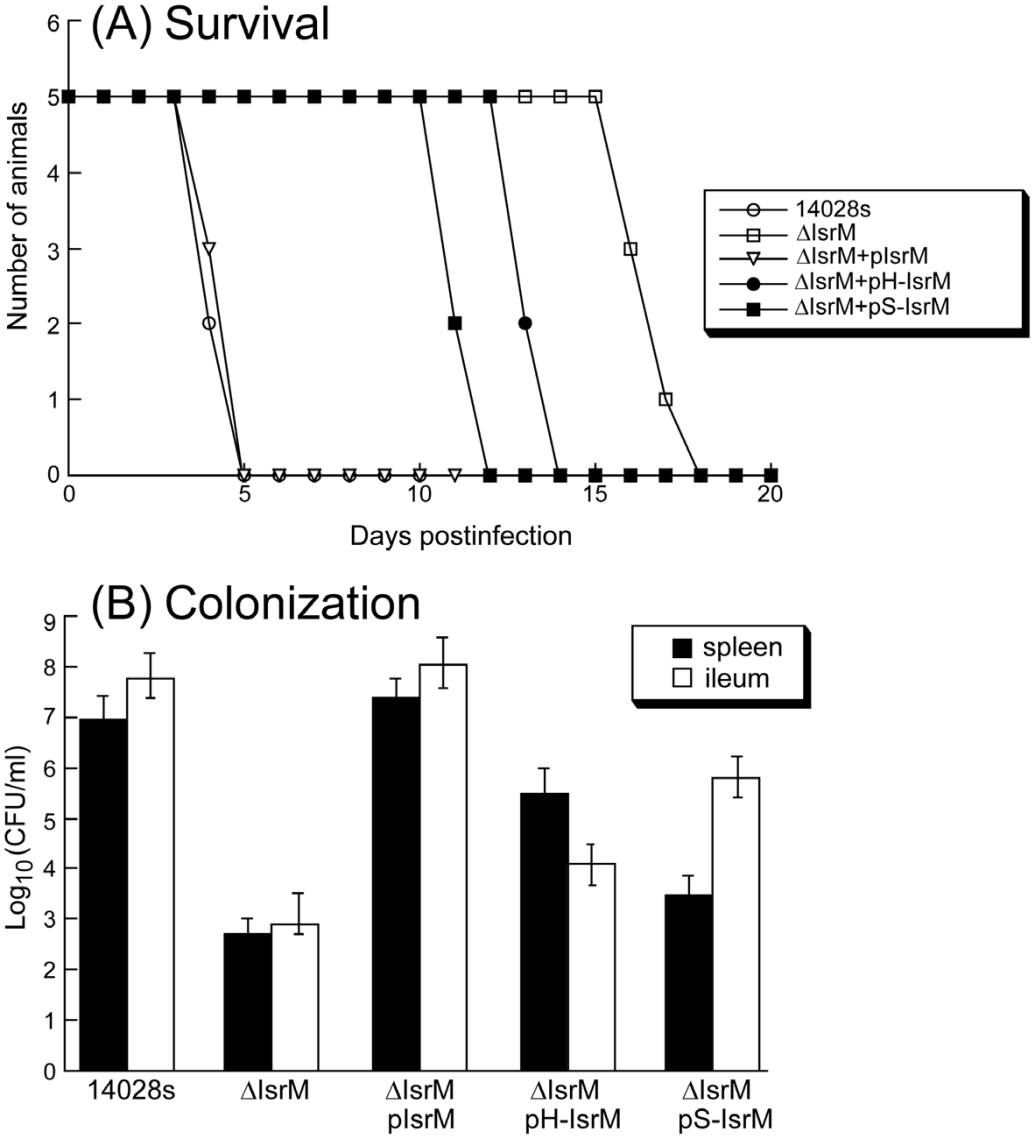
Virulence and colonization of *Salmonella* in mice. (A) Mortality of the BALB/c mice infected with isogenic strains carrying different constructs. BALB/c mice (5 animals per group) were infected intragastrically with *Salmonella* (5×10^6^ CFU). (B) The numbers of bacteria (CFU) in spleen and ileum of the infected animals. Groups of BALB/c (5 animals per group) mice were infected intragastrically (IG) with 1×10^5^ CFU of isogenic strains carrying different constructs, and bacteria were recovered from the organs at 7 days post inoculation. Each sample was analyzed in triplicate and the analysis was repeated at least three times. The CFU of the sample was expressed as the average of the values obtained. The concentrations of bacteria were recorded as CFU/ml of organ homogenate. The limit of bacteria detection in the organ homogenates was 10 CFU/ml.

To determine if IsrM is important for pathogenesis *in vivo*, BALB/c mice were infected intragastrically with the constructed *isrM* mutants. To study the virulence of *Salmonella*, the survival rates of the infected animals were determined. When mice were infected intragastrically with 5×10^6^ CFU bacteria, all animals inoculated with the wild type ST14028s strain and ΔIsrM carrying complementation construct pIsrM died within 5 days postinfection (Figure 7A). In contrast, mice infected with ΔIsrM, and ΔIsrM carrying construct pH-IsrM and pS-IsrM remained alive until 15, 12, and 10 days postinfection, respectively. Similar results were also observed in SCID mice that were intragastrically infected with these *Salmonella* strains (Figure S3)(Supporting Information). These results suggest that the reduced virulence of ΔIsrM is due to the deletion of IsrM and that IsrM is important for *Salmonella* virulence *in vivo.*

To further study the pathogenesis of *Salmonella* mutants in these animals, colonization of the mutants in the spleen and ileum was studied during a 7-day infection period before the onset of mortality of animals infected with the wild type ST14028s strain. At 7 days postinfection, the level of colonization of ΔIsrM carrying construct pIsrM was similar to that of ST14028s in the organs examined (Figure 7B). In contrast, the counts of ΔIsrM in spleens and ileums of the infected animals were lower than those of the ST14028s strain by approximately 15,000 and 70,000 fold, respectively (Figure 7B). The presence of construct pH-IsrM and pS-IsrM, which contained the mutated IsrM sequences with mutations that disrupted the interactions of IsrM RNA with the *hilE* and *sopA* mRNAs, respectively, did not completely restore the ability of mutant ΔIsrM to colonize these mice. Similar results were also observed in SCID mice that were intragastrically infected with these *Salmonella* strains (Figure S3)(Supporting Information). These results suggest that the attenuated colonization of ΔIsrM in these organs is probably due to the disruption of *isrM* and that IsrM sRNA may be required for optimal colonization and growth of *Salmonella* in these organs in mice.

## Discussion

### Pathogenicity island-encoded sRNA as a virulence factor

In this report, we presented the first direct evidence that IsrM modulates the expression of HilE and SopA proteins, and that its function is important for *Salmonella* invasion of epithelial cells, intracellular replication inside macrophages, and virulence and colonization in mice. A significant number of the sRNAs identified in *Salmonella* are encoded in the pathogenicity islands, which are not present in commensal bacteria or *E.coli* [23], [24], [25]. Only two of these sRNAs, InvR and IsrJ, have been characterized in detail. InvR, encoded in the SPI-1 region, represses outer membrane protein synthesis of *Salmonella* by regulating chromosomal genes and does not target any genes in the SPIs [24]. IsrJ, whose gene is located between STM2614 and STM2616, is expressed in the presence of the major SPI-1 transcription factor, HilA, and an *isrJ*-deletion mutant is defective in host cell invasion and effector translocation *in vitro* [23]. However, the targets of IsrJ sRNA have not been identified. Characterization of the mutants with inactivating InvR and IsrJ RNAs in animals has not been reported. The roles of either InvR or IsrJ sRNAs in bacterial pathogenesis and virulence in mice have not been determined.

Our results showed that IsrM sRNA targets the mRNAs of HilE and SopA independently, leading to down-regulation of expression of these two proteins. IsrM was expressed *in vitro* under conditions resembling those during the initiation of infection in the gastrointestinal tract (e. g. low pH, oxygen limitation, and high osmolarity). Furthermore, IsrM was found to be differentially expressed *in vivo*, with higher expression in the ileum than in the spleen. Mutations in IsrM resulted in disregulation of expression of HilE and SopA, as well as other SPI-1 genes whose expression is regulated by HilE. *Salmonella* with a deletion of *isrM* was defective in invasion of epithelial cells and intracellular replication/survival in macrophages, and the defect could be corrected by introduction of a complementation construct containing the full length *isrM* sequence. Moreover, *Salmonella* with mutations in *isrM* was attenuated in its virulence in killing animals and was defective in colonization in the ileum and spleen *in vivo*. These results suggest that IsrM sRNA encodes a virulence factor important for bacterial infection and demonstrate that a pathogenicity island-encoded sRNA is directly involved in *Salmonella* pathogenesis in mice. Our study raises the possibility that additional pathogenicity island-encoded sRNA may function in different aspects of *Salmonella* pathogenesis, including invasion of epithelium and intracellular replication in phagocytes.

### Differential expression of SPI-1 proteins regulated by sRNA

The regulation of expression of genes encoding SPI-1 proteins and its effectors is remarkably complex. For example, the transcription of many SPI-1 genes can be regulated by HilA, HilC, HilD, HilE, and PhoP/Q [8]. HilE encodes a global regulator that negatively modulates the expression of SPI-1 genes by sequestering HilD, which is a major transcriptional activator required for expression of most of the SPI-1 genes [35], [38]. Our results indicated that disruption of IsrM-mediated targeting of *hilE* mRNA results in increased expression of HilE protein and disregulated expression of SPI-1 proteins, leading to reduced invasion efficiency in cultured cells and attenuation of bacterial virulence and pathogenesis *in vivo*. Thus, bacterial sRNA presents another mechanism for global regulation of SPI-1 gene expression.

In addition to globally modulating the expression of SPI-1 proteins through HilE, IsrM also interacts with the mRNA of a specific SPI-1 effector, SopA. This interaction is likely to be important as IsrM-mediated modulation of SopA protein expression affects bacterial intracellular replication in macrophages (Figure 6). SopA has been shown to function as an E3 ubiquitin ligase regulating host inflammatory responses [39], [40]. The level of this protein is highly regulated *in vivo* as SopA is found to be expressed during the early and late stage of *Salmonella* infection *in vivo*, consistent with its role in invasion as well as in intracellular replication/survival [9], [12], [13]. Appropriate level of expression of SopA protein in different cells and at specific time points may contribute to defined consequences of pathogenesis. This is consistent with the recent observations that SopA exerts its functions in concert with other effector proteins during *Salmonella* infection of non-phagocytic cells; however, in the context of systemic infection, its ubiquitin ligase activity may facilitate bacterial replication in phagocytes [39], [41], [42]. IsrM can potentially negatively regulate SopA protein level at the translation level through its direct binding of the *sopA* mRNA and positively at the transcriptional level simultaneously through its down-regulation of the negative regulator HilE. This scenario may represent a novel mechanism of precise control of bacterial protein levels in a temporal and tissue/cell specific manner as required for pathogenesis.

It is currently unclear why IsrM is found in some serovars of *Salmonella* but not others. It is possible that the presence of IsrM in a specific serovar contributes to host specificity in a particular host. This issue can be investigated further by studying the presence of IsrM in different serovars and its role in the pathogenesis of different *Salmonella* strains in specific hosts *in vivo*. Our results indicated that IsrM binds to the 5′ UTR regions near the Shine-Dalgarno (SD) sequence of both the *hilE* and *sop*A mRNAs and inhibits the expression of these two proteins. While it is generally believed that binding of the SD region by sRNA results in translation repression, the exact mechanism of how IsrM reduces the expression of HilE and SopA proteins is currently unknown. Equally elusive is whether the IsrM-mediated reduction is mediated by bacterial proteins. The Sm-like RNA-binding protein Hfq, which has been found to be potentially associated with more than 40 *Salmonella* sRNAs [25], acts as a RNA chaperone that modulates the intracellular stability of many small non-coding RNAs and their annealing with target mRNAs for translation repression or stimulation [17], [43], [44]. It is unclear whether IsrM RNA is associated with or bound to Hfq, based on recent co-immunoprecipitation experiments [23], [25]. The SopA mRNA was found to be enriched moderately by co-immunoprecipitation with anti-Hfq antibodies while the HilE mRNA was not detected in these experiments [25]. Further studies are needed to characterize the molecular mechanism of the regulation mediated by IsrM.

It is generally believed that the amounts of various *Salmonella* SPI-1 proteins expressed *in vivo* are in a delicate balance as there are hierarchical transports of different effectors during *Salmonella* entry and extensively ordered synergistic and antagonist relationships between these effectors following their delivery into host cells [3], [45], [46]. It is conceivable that the ability of the bacteria to establish successful infection and cause pathogenesis in specific tissues may be significantly influenced by the balance of the amounts of these factors during infection. IsrM was found to be expressed *in vitro* under conditions resembling those during the initial infection in the gastrointestinal tract. Moreover, IsrM was found to be differentially expressed *in vivo*, with expression levels higher in the ileum than that in the spleen. Thus, controlled expression of IsrM is likely a mechanism *Salmonella* uses to achieve both global and specific regulation of the expression of SPI-1 proteins *in vivo* at the appropriate time and place in the host. It would be interesting to determine the expression patterns of IsrM as well as other sRNAs and their potential target mRNAs in different tissues and at different stages of infection *in vivo*, and study how their expression affects *Salmonella* infection. Further characterization of these sRNAs should provide significant insights into their exact roles in *Salmonella* infection and pathogenesis.

## Materials and Methods

### Ethics statement

This study was carried out in strict accordance with the recommendations in the Guide for the Care and Use of Laboratory Animals of the National Institutes of Health. The protocol for all animal experiments was approved by the Animal Care and Use Committee of the University of California-Berkeley (Protocol #R240 and #R276). All efforts were made to minimize suffering.

### Generation of *Salmonella* mutants and constructs

Table 1 lists the bacterial strains while Table 2 and 3 list the plasmid constructs and the primers used in the study, respectively. *Salmonella* strain ΔIsrM was derived from *S. typhimurium* strain ST14028s by deleting *isrM* with the λ Red recombinase method [47], following the procedures described previously [48]. Briefly, primers P5ΔIsrM and P3ΔIsrM (Table 3) were used to amplify the kanamycin resistance gene sequence in plamsid pKD4. The resulting PCR products were electrophorated into ST14028s carrying plasmid pKD46. Mutants undergoing homologous recombination were selected from electroporated ST14028s and confirmed by PCR using primers P5IsrM and P3IsrM. Once the deletion mutation was confirmed, the mutation was introduced into fresh culture of ST14028s by transduction using phage P22, and P22-free colonies were selected. The non-polar strain ΔIsrM was constructed using plasmid pCP20 [47], selected for its sensitivity to kanamycin, and further confirmed using PCR with the primers P5IsrM and P3IsrM (Table 3) [12], [13], [48].

**Table 2:** Plasmid constructs used in the study.

Plasmids	Description	Reference/source
pKD4	Containing a kanamycin resistance cassette and the flipase recognition sites	[47], [48]
pkD46	ApR, containing the Red recombinase of λ phage	[47]
pCP20	ApR, containing the expression cassette of flipase	[47]
pUC-H1PF1	ApR and KanR, template plasmid for 1xFLAG epitope tag	[49]
pBR322	ApR, cloning vector, backbone plasmid for constructing sRNA expression complementation plasmids	New England Biolab
pUC18	ApR, cloning vector	Invitrogen
pHG101	ApR; pBR322 with the multiple cloning sequence replaced by that of pUC18	This study
pIsrM	ApR, pHG101 containing *isrM* sequence for complementation of ΔIsrM mutant	This study
pH-IsrM	ApR, pIsrM with base substitutions (T_264_T_265_T_266_ ->A_264_A_265_G_266_) at the HilE mRNA binding site of the *isrM* sequence	This study
pS-IsrM	ApR, pIsrM with base substitutions (T_165_C_166_A_167_ ->A_165_G_166_T_167_) at the SopA mRNA binding site of the *isrM* sequence	This study
pZE12-luc	ApR, cloning vector for sRNA expression in *E.coli*	Expressys (Germany)
pLaco-IsrM	ApR, IsrM expression plasmid for validating the target genes of IsrM in *E.coli*	This study
pLaco-H-IsrM	ApR, pLaco-IsrM derivative containing nucleotide substitutions (T_264_T_265_T_266_ ->A_264_A_265_G_266_) at the HilE mRNA binding site of the *isrM* sequence	This study
pLaco-H2-IsrM	ApR, pLaco-IsrM derivative containing nucleotide substitutions (A_257_G_258_C_259_ ->T_257_C_258_G_259_) at the HilE mRNA binding site of the *isrM* sequence	This study
pLaco-S-IsrM	ApR, pLaco-IsrM derivative containing nucleotide substitutions (T_165_C_166_A_167_ ->A_165_G_166_T_167_) at the SopA mRNA binding site of of the *isrM* sequence	This study
pLaco-S2-IsrM	ApR, pLaco-IsrM derivative containing nucleotide substitutions (T_159_C_160_A_161_ ->A_159_G_160_T_161_) at the SopA mRNA binding site of of the *isrM* sequence	This study
pXG10	ChlR, expression plasmid for candidate target genes of IsrM in *E.coli*	[36]
pFG10HilEp1	ChlR, pXG10-derivative expression plasmid with the FLAG epitope fused to the 3′ end of the full-length HilE from the promoter 1	This study
pFG10HilEp2	ChlR, pXG10-derivative expression plasmid with the FLAG epitope fused to the 3′ end of the full-length HilE from the promoter 2	This study
pFG10HilEp3	ChlR, pXG10-derivative expression plasmid with the FLAG epitope fused to the 3′ end of the full-length HilE from the promoter 3	This study
pFG10-m-HilE	ChlR, pFG10HilEp1 derivative containing nucleotide substitutions (G_-3_A_-2_A_-1_ ->T_-3_T_-2_T_-1_) at the IsrM binding site of the *hilE* sequence	This study
pFG10-m2-HilE	ChlR, pFG10HilEp1 derivative containing nucleotide substitutions (G_4_C_5_T_6_ ->C_4_G_5_A_6_) at the IsrM binding site of the *hilE* sequence	This study
pFG10SopA	ChlR, pXG10-derivative expression plasmid with the FLAG sequence fused to the 3′ end of the *sopA* sequence from -61 nt to +774 nt.	This study
pFG10-M-SopA	ChlR, pFG10sopA derivative containing nucleotide substitutions (T_-21_G_-20_A_-19_ ->A_-21_C_-20_T_-19_) at the IsrM binding site of the *sopA* sequence	This study
pFG10-M2-SopA	ChlR, pFG10sopA derivative containing nucleotide substitutions (T_-15_G_-14_A_-13_ ->A_-15_C_-14_T_-13_) at the IsrM binding site of the *sopA* sequence	This study

**Table 3:** Primers used in the study.

Name	Sequence
P5qPCR-IsrM	5′-CACTTTGCGACTGTTCG-3′
P3qPCR-IsrM	5′-GGTGGTGATGATGTTGATATG-3′
P5qPCR-16S	5′-CGGGGAGGAAGGTGTTGTG-3′
P3qPCR-16S	5′-AGCCCGGGGATTTCACATC-3′
P5ΔIsrM	5′-TATCAAGCCTTTATCATTTTAAACTGAATTACGGTAGGCGTACCAACTTTGTATAAGATACATATGAATATCCTCCTTAGTTC-3′
P3ΔIsrM	5′-CTCATTCAGGGTGCCATAACTCGTAGTTCTCAGCAATTCTCACTGGACGACAATAGACGTTGTGTAGGCTGGAGCTGCTT-3′
P5IsrM	5′-TGTCCATTTAGTCACCATTACT-3′
P3IsrM	5′-AGCTTCTTAGCGATTTTTGCCA-3′
P5comp	5′-CCGGAATTCGGAAAAATGTTTTGATAAGTTGA-3′
P3comp	5′-CGCGGATCCTTCTTAGTGGGTCTCATTCAG-3′
P5b-Laco-IsrM	5′-CCGGAATTCTCTAGAGGCATCAAATAAAACG-3′
HilEFLAGu	5′-CCCGCATTAGCGTCGAAAAGCAAAACACGGCGGCCTCTTCACCGACAGGCGCTGTGGCGAGACTACAAAGACCATGACG-3′
HilEFLAGd	5′-GCTAAATGCCATTTCGCTATACAGCATCGCCCACTGCGAGTCCGCAAGCTTGTTTTGTCCCATATGAATATCCTCCTTAGTT-3′
HilEu	5′-CACCAATGTGCGAATTTCAC-3′
HilEd	5′-GCGGCTATGCGTAAAAATCA-3′
P3b-Laco-IsrM	5′-GTGCTCAGTATCTTGTTATCC-3′
P5i-Laco-IsrM	5′-p-AGCTTCATGCTTCTTTTTTAGAT-3′
P3i-Laco-IsrM	5′-CCGGAATTCTTCTTAGTGGGTCTCATTCAG-3′
P5i-fg10HilEp1	5′-AAAACTGCAGAATCCAGTTATAGCAGATTGTC-3′
P5i-fg10HilEp2	5′-AAAACTGCAGCAGGAAACGATGCTTGAATG-3′
P5i-fg10HilEp3	5′-AAAACTGCAGATAGTAAATATGTTCTATTGGAAT-3′
P3i-fg10HilEp1	5′-GCTCTAGATTACTTATCGTCGTCATCCTTGTAATCTCGCCACAGCGCCTGTCG-3′
P5i-fg10SopA	5′-AAAACTGCAGTACTTTTAAGGCGTTAAAAATCC-3′
P3i-fg10SopA	5′-GCTCTAGATTACTTATCGTCGTCATCCTTGTAATCGCAGACTACATCACTCAGTG-3′
P5i-Laco-H-IsrM	5′-p-TAGTTGTTTGAAGTTCAATGAAG-3′
P3b-Laco-H-IsrM	5′-CTTAGTGGCTGGCGTCCTTGTA-3′
P5i-Laco-H2-IsrM	5′-p-GTTTGAAGTTCAATGAAGTCT-3′
P3b-Laco-H2-IsrM	5′-TTGTAAAAAGTGGCTGGCGTCCT-3′
P5i-FG10-m-HilE	5′-AAAACTGCAGAATCCAGTTATAGCAGATTGTCGGTATTTAATCTGGTATACAGAGACACCAACTTTATGGCTGGAAAATGGAACGTT-3′
P5i-FG10-m2-HilE	5′-AAAACTGCAGAATCCAGTTATAGCAGATTGTCGGTATTTAATCTGGTATACAGAGACACCTTGGAAATGGCTGGAAAATGGAAC-3′
P5i-Laco-S-IsrM	5′-p-TCACCACCCTTTTTGCGCCT-3′
P3b-Laco-S-IsrM	5′-ACTTGTTGATATGGAAGGGGTTTTC-3′
P5i-Laco-S2-IsrM	5′-p-ACATCATCACCACCCTTTTTG-3′
P3b-Laco-S2-IsrM	5′-ACTTATGGAAGGGGTTTTCGTATC-3′
P5i-FG10-M-SopA	5′-AAAACTGCAGTACTTTTAAGGCGTTAAAAATCCAGACCGTTTTTCCATAAACTTGTTGATAAGGAATTCTAATGAA-3′
P5i-FG10-M2-SopA	5′-AAAACTGCAGTACTTTTAAGGCGTTAAAAATCCAGACCGTTTTTCCATAATGATGTACTTAAGGAATTCTAATGAAGATATCA-3′

Mutant T-hilE was constructed using the λ Red recombinase method [47], following the procedures as described previously [12], [13], [48]. A pair of primers was designed to amplify the FLAG epitope coding sequence and kanamycin resistance gene using pUC-H1PF1 as the template [49]. The resulting PCR products were electroporated into *Salmonella* ST14028s strain carrying plasmid pKD46. The non-polar strain T-hilE was constructed using plasmid pCP20 [47], selected for its sensitivity to kanamycin, and further confirmed using PCR and subsequent DNA sequencing. To generate *Salmonella* strains bearing different tagged SPI-1 proteins and the *isrM*-deletion mutation, *Salmonella* SPI-1 protein-tagged non-polar strains T-invJ, T-sipA, T-sipB, T-sipC, T-sipD, T-sopA, T-sopB, T-sopE2, T-spaO, T-sptP, which were previously constructed in our laboratory [12], [13], were transduced with phage P22 carrying the *isrM*-deletion mutation, following the procedures described previously [12], [13]. Similar procedures were used to generate mutant ΔM-T-hilE. The regions for the tagged ORFs in all the generated tagged strains were sequenced to confirm that no other mutations were present in these regions [12], [13], [48].

To generate complementation plasmids to express the wild type and mutant IsrM RNA under the control of its native promoter, plasmid pBR322 was used as the backbone to generate construct pHG101. Construct pIsrM was generated by inserting to pHG101 with the IsrM sequence generated by PCR with primers P5comp and P3comp (Table 3) that annealed to a 20-nt sequence located at 208 nucleotides upstream from the transcription initiating site and 51 nucleotides downstream from the transcription terminator of IsrM, respectively.

### *In vitro* growth analysis of *Salmonella*

Growth analysis of bacteria in LB broth was carried out by first inoculating a single colony from a fresh plate in 2 ml LB broth and culturing at 37°C with shaking at 250 RPM overnight for at least 15 hours [12]. Thirty microliters of the overnight culture were then inoculated into 3 ml fresh LB broth and cultured at 37°C and 250 RPM. At time points of 0, 2, 4, 6, 8, 10, 12, 14, 16, and 24 hours after inoculation, 100 microliters of bacterial culture were collected and used for analysis by serial dilution in sterile 96-well plates, and then plated on LB agar plates to determine their CFU/ml. Each sample was analyzed in triplicates and the analysis was repeated at least three times. The average value of CFU/ml was used to generate the growth curve [12].

### Expression of IsrM sRNA *in vitro*

To study the effect of different culture conditions on the IsrM expression *in vitro*, 20 µl of overnight bacterial cultures were inoculated into 1 ml of LB broth and shaken at 250 RPM and 37°C for 4 hours. The bacterial cultures were centrifuged at 5,000xg for 5 minutes. To study the effect of pH, the pelleted bacteria were re-suspended in 1 ml fresh LB broth (control, pH7.0) or 1 ml LB broth at pH 3.0, 4.4, 5.4, 6.4, 7.2, or 8.4, respectively, shaken at 250 RPM and 37°C for an additional 3 hours, and then collected. To study the effect of osmolarity, the pelleted bacteria were re-suspended in 1 ml NaCl-free LB broth supplemented with 0, 42.5, 85, 160, 340, and 680 mM sodium chloride, respectively, shaken at 250 RPM and 37°C for an additional 3 hours, and were collected. Regular LB broth, which contained 170 mM NaCl, was used as the control. To study the effect of oxygen limitation, the pelleted bacteria were re-suspended in 1.5 ml fresh LB broth. One group of bacteria was shaken at 250 RPM and 37°C for an additional 3 hours with good aeration (control) while another group of bacteria was transferred into 1.5 ml microcentrifuge tubes with their covers closed tightly and wrapped with parafilm, and incubated at 37°C without shaking for an additional 3 hours. Bacteria were pelleted by centrifugation (2 min, 13,000 rpm, 4°C), and RNA samples were isolated with the Trizol method (Invitrogen, Carlsbad, CA), treated with DNase I (TURBO DNA-*free*, Ambion, Austin, TX), and used for quantitative RT-PCR analysis, following the procedures described previously [13], [48], [50]. The analyses were repeated three times and the average of three experiments are presented. For Northern blot analysis, the *Salmonella* RNA samples were separated in 2% agarose gels that contained formaldehyde, transferred to nitrocellulose membranes, hybridized with the [^32^P]-radiolabeled DNA probes that contained the DNA sequence coding for *Salmonella* IsrM and 5S RNA, and analyzed with a STORM840 Phosphorimager [49].

### Validation of IsrM targets in *E.coli*

The *TargetRNA* and *RNAhybrid* programs were used to predict the target mRNAs of IsrM against the entire genome of *Salmonella typhimurium* ST14028s [33], [34], and the RNAup software (http://rna.tbi.univie.ac.at/cgi-bin/RNAup.cgi) was used to analyze the potential interaction of IsrM to the *hilE* and *sopA* mRNAs. Constructs to validate the targets of IsrM in *E.coli* were generated following a modified procedure described previously [36]. To generate construct pLaco-IsrM expressing IsrM, a 2.2 kb DNA fragment, which contained a PLlacO promoter (from the position -1), an ampicillin resistance cassette, a ColE1 replicon, and a strong *rrnB* terminator, was amplified from the high-copy plasmid, pZE12luc (EXPRESSYS, Ruelzheim, Germany), with primers P5b-Laco-IsrM and P3b-Laco-IsrM. This DNA fragment was used as the backbone to ligate with the DNA fragment (generated in PCR with primers P5i-Laco-IsrM and P3i-Laco-IsrM) containing the entire IsrM coding sequence and its transcription terminator sequence, generating construct pLaco-IsrM.

To generate constructs expressing HilE and SopA proteins in *E.coli*, the low-copy plasmid, pXG10 (a gift from Dr. J. Vogel, Germany) [36], was used as the backbone to insert the *hilE* and *sopA* sequence for expression of 1×FLAG fusion proteins. The entire coding sequence of *hilE* was included in the FLAG-fusion protein. As *hilE* has three initiating sites of transcription, three plasmid constructs (pFG10HilEp1, pFG10HilEp2, and pFG10HilEp3) were generated to express the HilE-FLAG fusion protein. Since the transcription initiation site of the *sopA* mRNA has not definitively been determined, construct pFG10SopA was generated to contain the sequence between the position 61 nt upstream the translation initiation codon and the position coding for the 258^th^ amino acid residue of SopA, a protein of 782 amino acids [51]. Constructs expressing mutated *isrM* and its target genes carrying different mutations (e.g. pLaco-H-IsrM, pFG10-m-HilE, pLaco-S-IsrM, and pFG10-M-SopA) were generated by a PCR-based mutagenesis approach, using primers listed in Table 3.

### Expression of tagged proteins in bacteria

To study the expression of tagged proteins in *E.coli*, the plasmids for expressing HilE- and SopA-FLAG proteins (e.g. pFG10HilEp1, pFG10HilEp2, pFG10HilEp3, and pFG10SopA) were co-transformed into *E coli* Top10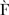 with pLaco-IsrM and its derivatives, which contained the wild type and mutated *isrM* sequence, respectively. The transformants expressed HilE- and SopA-FLAG proteins in the presence of 20 µg/ml tetracycline and expressed isrM and its related RNA in the presence of 1 mM IPTG. Plasmid pZE12lu (Table 2), which served as the backbone of RNA expression plasmids for target validation, was used as the control plasmid.

To determine the effect of the wild type and mutated IsrM RNA on the expression of HilE and SopA, 60 µl of overnight culture of *E.coli* Top10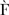 that contained both an expression plasmid for IsrM and an expression plasmid for SopA- or HilE-FLAG protein was inoculated in 3 ml of LB broth containing ampicillin (100 µg/ml) and chloramphenicol (20 µg/ml), and shaken at 37°C, 250 RPM for 4 hours. Tetracycline and IPTG were added into bacterial cultures to a final concentration of 20 µg/ml and 1 mM, respectively, and shaken at 37°C, 250 RPM for an additional six hours. Bacterial pellets were collected and protein samples were isolated. Western blot analysis was performed to determine the levels of the tagged proteins with the expression of GroEL as the internal control [13], [48], [50].

To study protein expression in *Salmonella*, the SPI-1 protein-tagged strains carrying the deletion mutation of *isrM* were inoculated in 2 ml of LB broth and grew at 37°C and 250 RPM overnight. Sixty microliters of overnight bacterial culture was inoculated in 3 ml of LB broth and grew at 37°C and 250 RPM for 6 hours. Protein samples were prepared and Western blot analysis was performed using the expression of DnaK as the internal control, following the procedure described previously [13], [48], [50]. The analyses were repeated three times.

### Western blot analyses

Polypeptides from bacterial lysates were separated on SDS-containing 10-12% polyacrylamide gels, transferred to nitrocellulose membranes, and reacted with anti-mouse IgG conjugated with alkaline phosphatase in addition to the antibodies against DnaK protein (StressGen, Victoria, BC, Canada), GroEL protein (Sigma, St Louis, MO), or the FLAG sequence (Sigma, St Louis, MO), following the procedure described previously [13]. The membranes were subsequently stained with a chemiluminescent substrate with the aid of a Western chemiluminescent substrate kit (GE Healthcare, Waukesha, WI) and quantified with a STORM840 phosphorimager. Normalization of samples was also carried out by loading total proteins extracted from the same CFU (e.g. 5×10^7^ CFU) of bacteria in each lane. Quantitation was performed in the linear range of protein detection.

### Invasion and intracellular replication assays

Assays for bacterial invasion in HeLa cells and intracellular survival/replication in J774 macrophages were performed as described previously [37], [49], [50]. Briefly, *Salmonella* was added to HeLa cells at multiplicities of infection (MOI) of 5:1 to 10:1, and intracellular bacteria were quantified after 1 h of incubation for invasion, which was followed by incubation in the presence of 50 µg/ml of gentamicin to kill extracellular bacteria. The invasiveness of *Salmonella* was measured by determining the ratio of intracellular bacteria, which was calculated as follows: (number of intracellular bacteria/number of input bacteria) ×100 [49], [50].

To study intracellular growth of *Salmonella* in macrophages, mouse J774 macrophages were infected with *Salmonella* for 1 h at an MOI of 10, washed with phosphate-buffered saline (PBS), and then incubated in fresh medium containing 50 µg/ml of gentamicin for 1 hour. A set of cells was washed and lysed to quantify the intracellular bacteria and used as the zero-time (0-h) sample. Additional sets of cells were harvested after an additional 8 h of incubation to quantify intracellular *Salmonella* (8-h samples). The growth of *Salmonella* inside J774 cells was measured by determining the increase in the number of intracellular *Salmonella*, which was calculated by dividing the number of intracellular bacteria in the 8-h sample by that in the zero-time sample [49], [50]. All analyses were repeated three times.

### Animal studies

Female BALB/c and SCID mice (6–8 weeks old) were obtained from Jackson Laboratory (Bar Harbor, ME). Overnight bacterial cultures were serially diluted to suitable colony forming unit (CFU)/ml in phosphate-buffered saline (PBS) for infection. To assess the virulence of the *Salmonella* strains, mice (5 animals per group) were either inoculated intragastrically with 5×10^6^ CFU per BALB/c mouse and 1×10^3^ CFU per SCID mouse or intraperitoneally with 1×10^2^ CFU per BALB/c mouse and 1×10^1^ CFU per SCID mouse. The survival of the infected animals was monitored and the survival rate was recorded.

For IsrM sRNA expression and organ colonization experiments, mice (5 animals per group) were inoculated intraperitoneally with 1×10^5^ or 1×10^7^ CFU per BALB/c mouse or 1×10^2^ or 1×10^4^ CFU per SCID mouse of the bacterial strains, and were euthanized at 7 days or 18 hours after inoculation, respectively. Mice (5 animals per group) were also inoculated intragastrically with 1×10^5^ or 1×10^8^ CFU per BALB/c mouse or 1×10^2^ or 1×10^4^ CFU per SCID mouse of the bacterial strains and were euthanized at 7 days or 18 hours after inoculation, respectively. Spleen was collected from infected mice, and ileum was dissected from the small intestine. All organs were homogenized in cold PBS, and bacterial CFU was determined by plating of serially diluted organ homogenates [13], [48], [50]. The analyses were repeated three times.

To determine the level of IsrM RNA, the homogenates of the spleen and ileum samples were centrifuged at 9,000xg at 4°C for 10 minutes [13], [48], [50]. Pellets were incubated in lysis buffer (120 mM NaCl, 4 mM MgCl_2_, 20 mM Tris/HCl, pH 7.5, 1% Triton-X100) at 4°C for 1 hour, and released bacteria were collected by centrifugation at 18,000xg for 10 minutes. Harvested bacteria were then resuspended in PBS, and adjusted for the bacterial CFU. RNA samples were prepared from isolated *Salmonella* and analyzed by qRT-PCR to determine the expression of IsrM RNA [13], [48], [50]. The analyses were repeated three times.

## Supporting Information

Figure S1 The levels of IsrM in *Salmonella* grown in vitro at pH4.4, pH7.0, and pH7.2, as determined by Northern blot analyses. The levels of *Salmonella* 5S RNA were used as the internal control. The experimental procedures are described in Materials and Methods. The *Salmonella* RNA samples were separated in 2% agarose gels that contained formaldehyde, transferred to nitrocellulose membranes, hybridized with the [^32^P]-radiolabeled DNA probes that contained the DNA sequence coding for *Salmonella* IsrM and 5S RNA, and analyzed with a STORM840 Phosphorimager.
(TIF)

Figure S2 Growth analysis of *Salmonella* strains in LB broth. (A) The growth of the wild type ST14028s, mutant ΔIsrM, and ΔIsrM transformed with different plasmids. (B) The growth of the wild type ST14028s and the SPI-1 protein-tagged *Salmonella* strains. The experimental procedures are described in Materials and Methods. The results are the means of three experiments performed in triplicate. The error bars indicate standard deviations.
(TIF)

Figure S3 Virulence and colonization of *Salmonella* in mice. (A) Mortality of the SCID mice infected with isogenic strains carrying different constructs. SCID mice (5 animals per group) were infected intragastrically with *Salmonella* (1×10^3^ CFU). (B) The numbers of bacteria (CFU) in spleen and ileum of the infected animals. Groups of SCID (5 animals per group) mice were infected intragastrically (IG) with 1×10^2^ CFU of isogenic strains carrying different constructs, and bacteria were recovered from the organs at 7 days post inoculation. Each sample was analyzed in triplicate and the analysis was repeated at least three times. The CFU of the sample was expressed as the average of the values obtained. The concentrations of bacteria were recorded as CFU/ml of organ homogenate. The limit of bacteria detection in the organ homogenates was 10 CFU/ml.
(TIF)

Table S1 Relative levels of IsrM in *E. coli* transformed with different constructs, as compared to those in *E. coli* that expressed HilE-FLAG and IsrM in the presence of IPTG. Relative levels of HilE protein in *E. coli* transformed with different constructs, as compared to those in the pIsrM-containing *E. coli* that expressed HilE-FLAG and m-HilE-FLAG in the absence of IPTG, respectively.
(DOC)

Table S2 Relative levels of IsrM in *E. coli* transformed with different constructs, as compared to those in *E. coli* that expressed SopA-FLAG and IsrM in the presence of IPTG. Relative levels of SopA protein in *E. coli* transformed with different constructs, as compared to those in the pIsrM-containing *E. coli* that expressed SopA-FLAG and M-SopA-FLAG in the absence of IPTG, respectively.
(DOC)
