# Supplementary figures and images for: A Salmonella Small Non-Coding RNA Facilitates Bacterial Invasion and Intracellular Replication by Modulating the Expression of Virulence Factors
Source: PLoS Pathog. 2011 Sep 15;7(9):e1002120. doi: 10.1371/journal.ppat.1002120 (PMC3174252; doi:10.1371/journal.ppat.1002120)

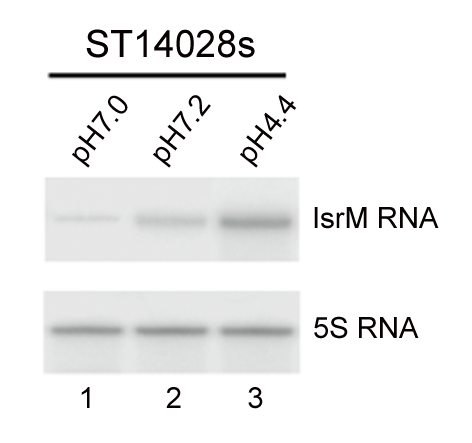

Supplement: Figure S1 — The levels of IsrM in Salmonella grown in vitro at pH4.4, pH7.0, and pH7.2, as determined by Northern blot analyses. The levels of Salmonella 5S RNA were used as the internal control. The experimental procedures are described in Materials and Methods. The Salmonella RNA samples were separated in 2% agarose gels that contained formaldehyde, transferred to nitrocellulose membranes, hybridized with the [32P]-radiolabeled DNA probes that contained the DNA sequence coding for Salmonella IsrM and 5S RNA, and analyzed with a STORM840 Phosphorimager. (TIF) [file ppat.1002120.s001.tif]

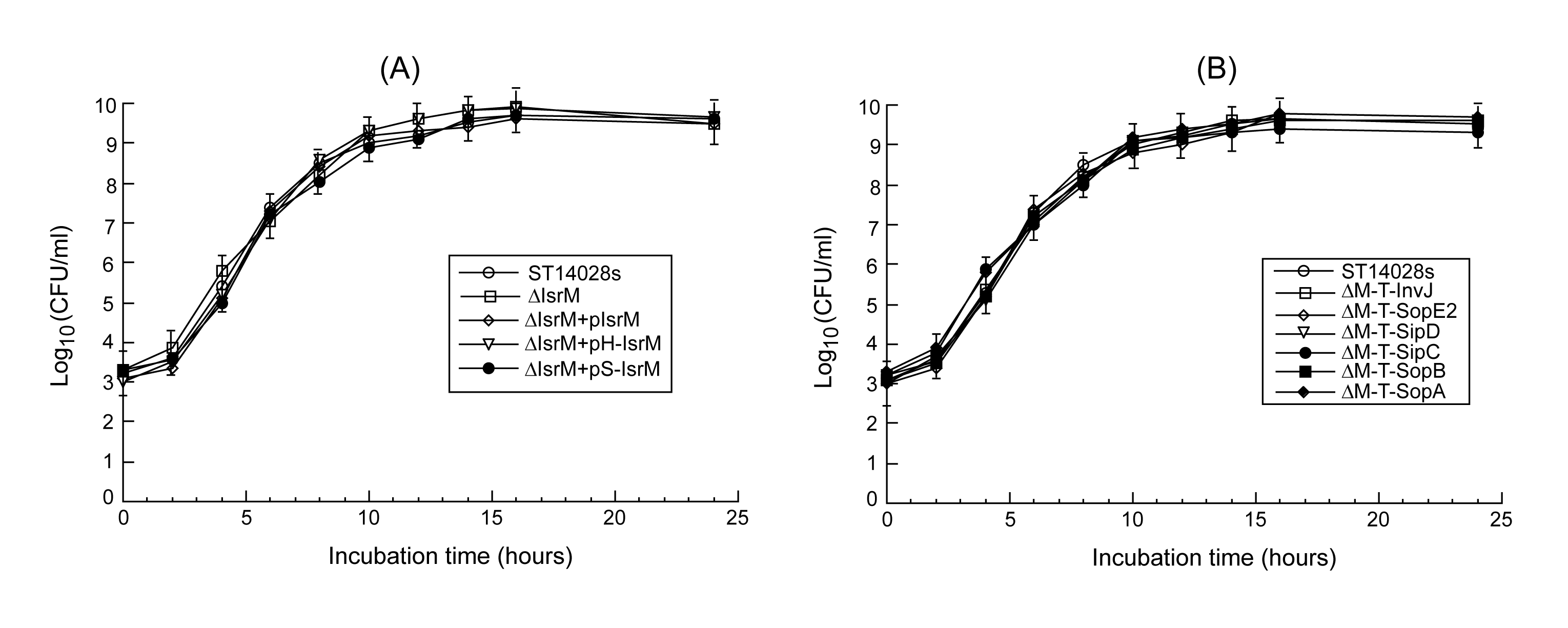

Supplement: Figure S2 — Growth analysis of Salmonella strains in LB broth. (A) The growth of the wild type ST14028s, mutant ΔIsrM, and ΔIsrM transformed with different plasmids. (B) The growth of the wild type ST14028s and the SPI-1 protein-tagged Salmonella strains. The experimental procedures are described in Materials and Methods. The results are the means of three experiments performed in triplicate. The error bars indicate standard deviations. (TIF) [file ppat.1002120.s002.tif]

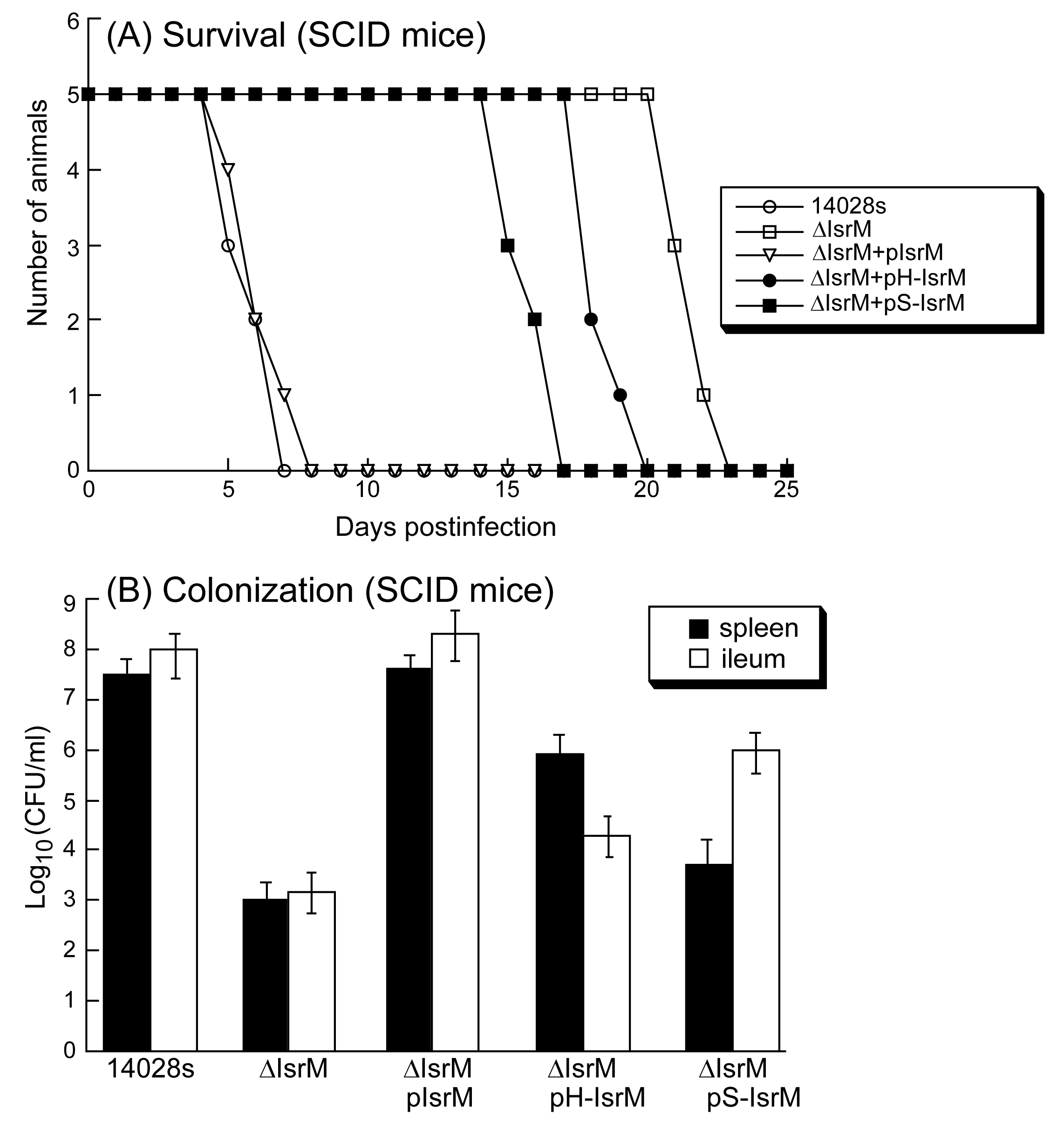

Supplement: Figure S3 — Virulence and colonization of Salmonella in mice. (A) Mortality of the SCID mice infected with isogenic strains carrying different constructs. SCID mice (5 animals per group) were infected intragastrically with Salmonella (1×10^3^ CFU). (B) The numbers of bacteria (CFU) in spleen and ileum of the infected animals. Groups of SCID (5 animals per group) mice were infected intragastrically (IG) with 1×10^2^ CFU of isogenic strains carrying different constructs, and bacteria were recovered from the organs at 7 days post inoculation. Each sample was analyzed in triplicate and the analysis was repeated at least three times. The CFU of the sample was expressed as the average of the values obtained. The concentrations of bacteria were recorded as CFU/ml of organ homogenate. The limit of bacteria detection in the organ homogenates was 10 CFU/ml. (TIF) [file ppat.1002120.s003.tif]
