# Supplementary material for: A Salmonella Small Non-Coding RNA Facilitates Bacterial Invasion and Intracellular Replication by Modulating the Expression of Virulence Factors
Source: PLoS Pathog. 2011 Sep 15;7(9):e1002120. doi: 10.1371/journal.ppat.1002120 (PMC3174252; doi:10.1371/journal.ppat.1002120)
Supplement: Table S2 — Relative levels of IsrM in E. coli transformed with different constructs, as compared to those in E. coli that expressed SopA-FLAG and IsrM in the presence of IPTG. Relative levels of SopA protein in E. coli transformed with different constructs, as compared to those in the pIsrM-containing E. coli that expressed SopA-FLAG and M-SopA-FLAG in the absence of IPTG, respectively. (DOC) [file ppat.1002120.s005.doc]

Table S2. Relative levels of IsrM in *E. coli* transformed with different constructs, as compared to those in *E. coli* that expressed SopA-FLAG and IsrM in the presence of IPTG. Relative levels of SopA protein in *E. coli* transformed with different constructs, as compared to those in the pIsrM-containing *E. coli* that expressed SopA-FLAG and M-SopA-FLAG in the absence of IPTG, respectively.

| Expressed Protein | SopA-FLAG | | | | M-SopA-FLAG | | | |
| --- | --- | --- | --- | --- | --- | --- | --- | --- |
| Expressed sRNA | IsrM | | S-IsrM | | IsrM | | S-IsrM | |
| IPTG | - | + | + | - | - | + | + | - |
| Level of IsrM | <5% | 100% | 99+9% | <5% | <5% | 101+9% | 96+11% | <5% |
| Level of SopA protein | 100% | 8+7% | 97+7% | 90+10% | 100% | 104+9% | 8+5% | 99+11% |

The values shown are the means of triplicate experiments. The levels of IsrM were determined by qRT-PCR, using the levels of 16S rRNA as the internal control. The levels of SopA protein were determined by Western blot analyses, using the levels of GroEL as the internal control.
